# P-1558. Protective Bile Acid Profiles in Immunocompromised Pediatric Patients with Confirmed CDI Toxin

**DOI:** 10.1093/ofid/ofaf695.1738

**Published:** 2026-01-11

**Authors:** Palak Patel, Nathalia A Davila, Taylor Abbey, Meghan Landry, David Zhang, Madan Kumar

**Affiliations:** The University of Chicago, Chicago, Illinois; University of Chicago, Chicago, Illinois; University of Chicago, Chicago, Illinois; University of Chicago, Chicago, Illinois; University of Chicago Medicine, Chicago, Illinois; University of Chicago Medicine, Chicago, Illinois

## Abstract

**Background:**

Oncologic patients are disproportionately affected by *Clostridioides difficile* (C. diff) infection (CDI) due to increased risk factors, including those undergoing hematopoietic stem cell transplantation (HSCT). We have previously shown that pediatric patients have both colonization with toxigenic C. diff isolates and measurable toxin production without evidence of clinical disease. We aim to prove that establishing metabolomic protection despite toxin presence we can introduce a new potential target for therapeutics and help resolve the substantial diagnostic uncertainty.

**Figure:**
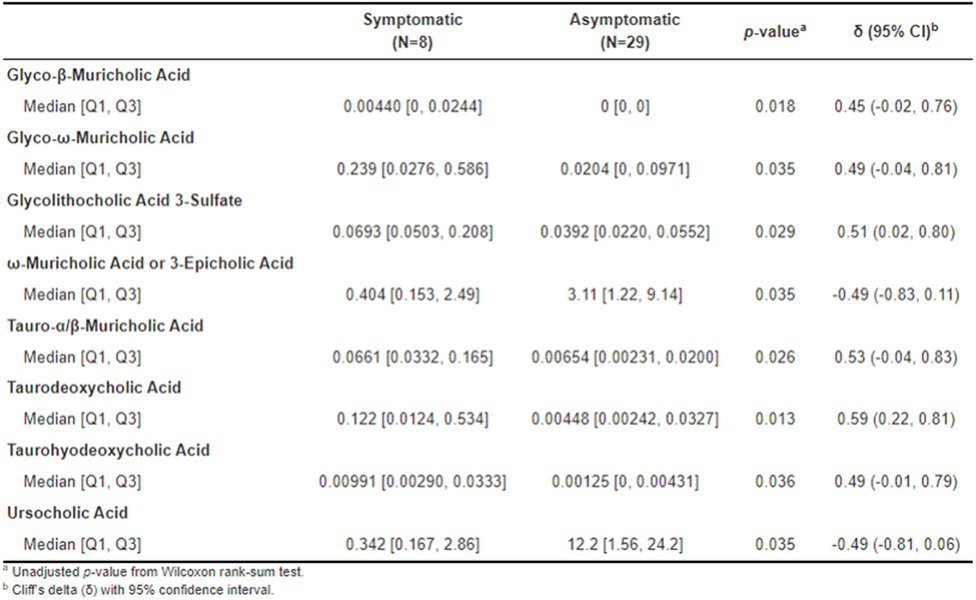
Summary of Stats

**Figure:**
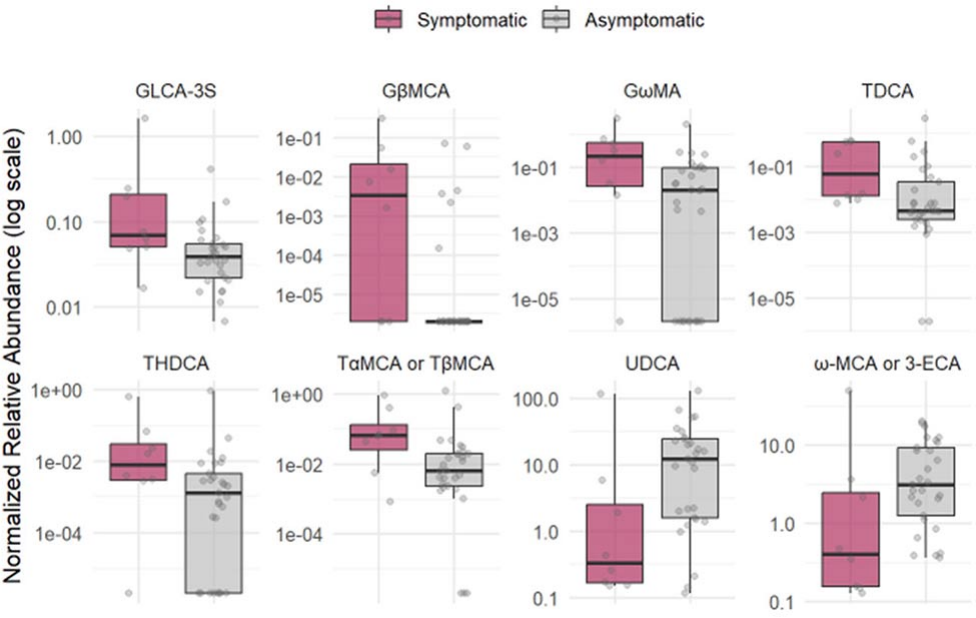
Box Plot of Bile Acids Bile acids comparisons of symptomatic vs asymptomatic toxin positive patients

**Methods:**

As part of a multi-center, prospective stool banking study, pediatric patients admitted with an oncologic diagnosis were enrolled between November 2019 to March 2023. A total of 98 participants were included and followed longitudinally for all subsequent inpatient admissions. Stool from children with CDI risk factors and surveys were collected weekly, and isolated C. diff were cultured, and Cell Cytotoxicity Neutralization Assay (CCNA) was performed. Following CCNA testing, bile acids were extracted from CCNA(+) stool using an organic solvent in beadruptor tubes for analysis by liquid chromatography-mass spectrometry. *P*-values were calculated using Wilcoxon rank-sum test with a significance threshold set at α = 0.05.

**Figure:**
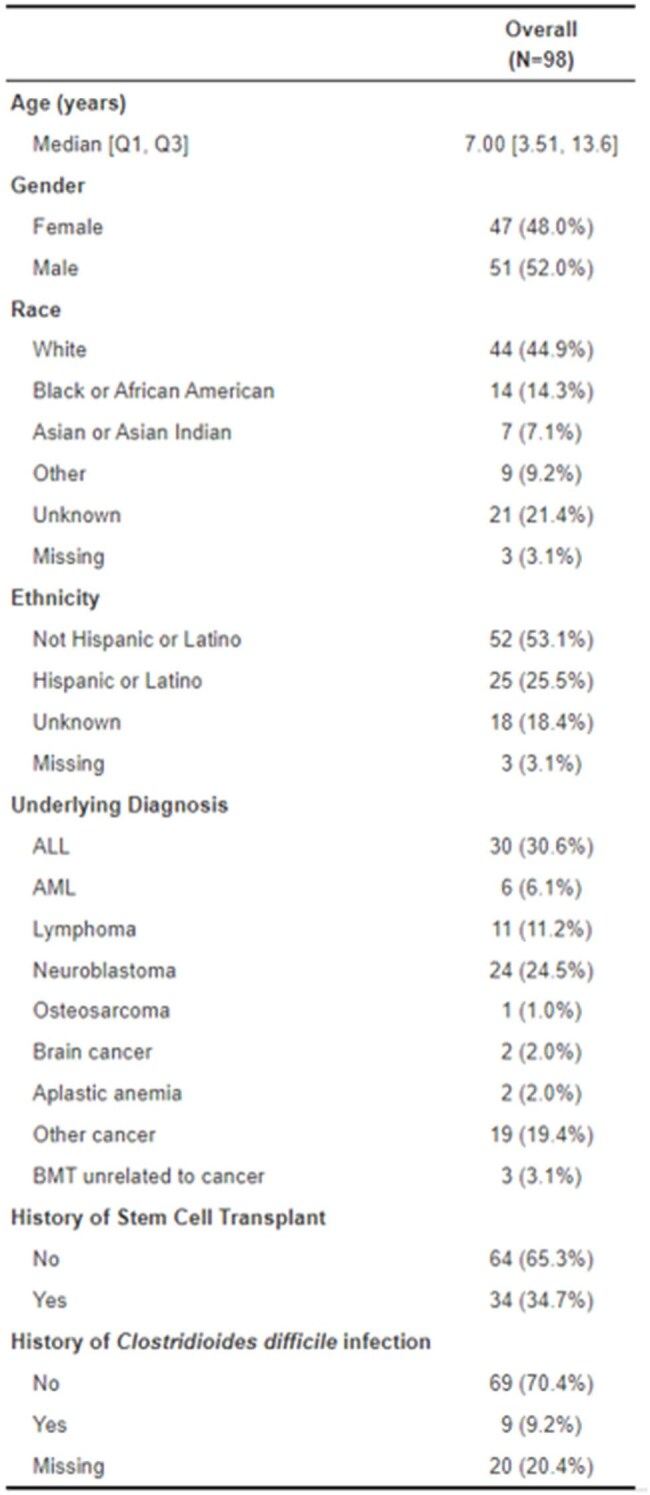
Baseline Characteristics

**Figure:**
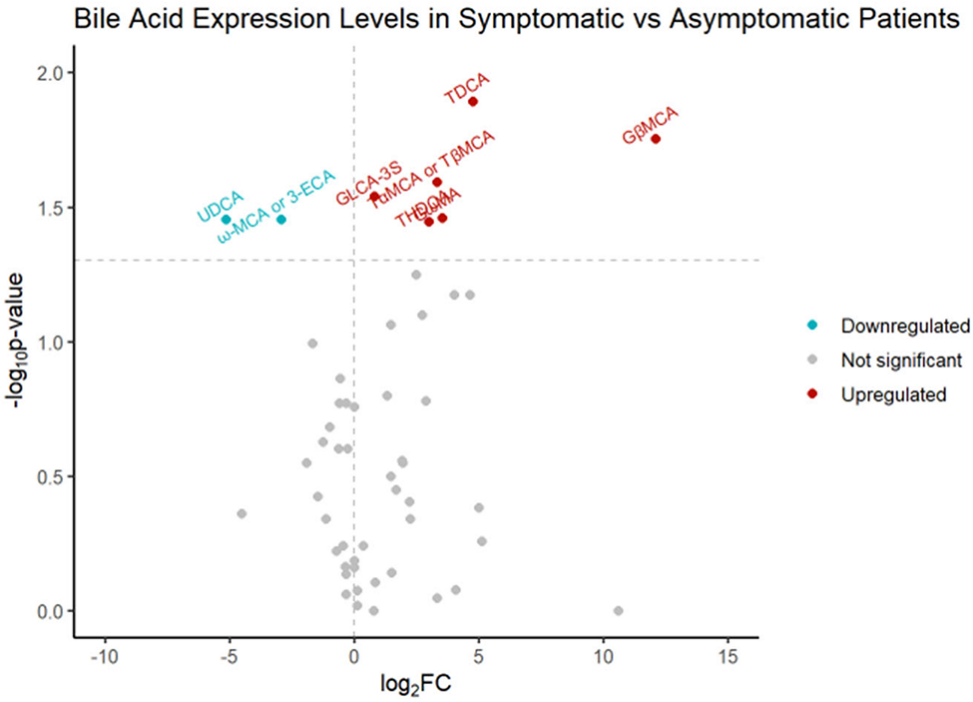
Volcano Plot

**Results:**

Of the 98 enrolled participants, the median age was 7 years (IQR: [3.5, 13.6]), and 45% and 52% were Caucasian and male, respectively. A total of 37 CCNA positive stool samples were sent for untargeted metabolomic profiling of bile acids. Of these, 78% of participants were asymptomatic at the time of collection. Eight bile acids were significantly up- or downregulated in the symptomatic cohort.

**Conclusion:**

These preliminary findings suggested 8 bile acids are significantly associated with CDI symptomology; future work should validate these trends in larger samples.

**Disclosures:**

All Authors: No reported disclosures

